# ZL006 Treatment Reduces Inflammation, Oxidative Stress, and Brain Aβ_1–42_ Accumulation and Rescues the Loss of PSD95 Synaptic Marker in Familial Alzheimer’s Disease-Associated *psen1*-Deficient Zebrafish Model

**DOI:** 10.3390/ijms27114992

**Published:** 2026-05-30

**Authors:** Serena Ricci, Maria Benuzzi, Martina Fazzina, Pietro Cacialli

**Affiliations:** Department of Biological, Geological and Environmental Sciences (BIGEA), University of Bologna, 40126 Bologna, Italymaria.benuzzi@studio.unibo.it (M.B.); martina.fazzina2@unibo.it (M.F.)

**Keywords:** zebrafish, Alzheimer’s disease, neuroprotection, ZL006

## Abstract

Familial Alzheimer’s disease (FAD) is a rare form of Alzheimer’s. FAD is mainly caused by one or multiple mutations in the genes encoding for amyloid precursor protein (APP), presenilin-1 (PSEN1), and presenilin-2 (PSEN2), with the majority occurring in PSEN1. Despite extensive research in animal models and numerous promising treatment trials, there is still no curative treatment for FAD. Recently, ZL006 (Med Chem Express cat. Number HY-100456) was shown to reduce over-produced nitric oxide and oxidative stress in ischemic stroke and could protect neurons against Aβ_1–42_-induced neurotoxicity (in vitro study). With this in mind, we tested ZL006 at different doses (10 μM, 25 μM, 50 μM and 100 μM) in zebrafish embryo injected with ctrl-MO and *psen1*-MO, investigating the effects on pathological phenotype in vivo. We showed that ZL006 exposure suppresses inflammation, oxidative stress and accumulation of Aβ_1–42_ in *psen1*-MO. In conclusion, our study showed that ZL006 was able to ameliorate the pathological phenotype of *psen1*-morphant zebrafish embryos, supporting its potential as a candidate for further investigations in the context of FAD treatment.

## 1. Introduction

Alzheimer’s disease (AD) is the most common neurodegenerative disease and can be divided into two subtypes: familial AD (FAD) and sporadic AD (SAD), which share similar clinical manifestations [1]. SAD is the most common form of dementia with an occurrence of over 95% in AD patients, characterized by a late onset (typically after age 65) without a direct inherited familial link. It is considered a multi-factorial disorder in which both genetic and environmental factors are involved in the genesis of the disease. On the other hand, FAD is a rare form of Alzheimer’s that is entirely dependent on genetics, being inherited from parents [1]. FAD differs from the more common late-onset Alzheimer’s [2] by its early onset (symptoms developing in people at 30 or 40 years old) and its rarity (5% of disease incidence), and it is not included in most clinical and drug trials [3]. FAD is caused by one or more autosomal dominant mutations in the gene that encodes for amyloid-β precursor protein (*APP*), presenilin1 (*PSEN1*) and or presenilin2 (*PSEN2*) [4]. The majority of FADs are caused by mutations in *PSEN1* [5] [6].

The earliest events that occur in this neurodegenerative disease [6] include a disruption of energy metabolism due to the abnormal accumulation of amyloid beta (Aβ), which ultimately leads to the dysfunction and death of neuronal cells and cognitive impairment [6,7,8,9]. In vitro and in vivo models of familial AD mutations have reported an increase in reactive oxygen species (ROS) production and a decrease in adenosine triphosphate (ATP) levels [10,11,12]. Moreover, hydrogen peroxide levels were found to be significantly increased and directly correlated with levels of soluble Aβ, and cytochrome-c oxidase (also known as COX-4) activity was found to be decreased [10,13,14]. Recent studies further indicate that AD is associated with increased neuronal nitric oxide synthase (nNOS) expression interacting with the protein postsynaptic density-95 (PSD-95), resulting in excessive nitric oxide (NO) production and consequent neuronal injury [15,16]. As with the more common late-onset Alzheimer’s, FAD has no cure and causes the person diagnosed to eventually become completely dependent on caregivers and require time assistance. In this respect, there is an urgent need to identify new potential therapeutic targets for this disease. ZL006, a potent inhibitor of nNOS/PSD-95 interaction [17], has been shown to exert neuroprotective effects across different experimental systems. A recent in vitro study in Aβ_1–42_-treated neuronal cells indeed demonstrated that ZL006 can attenuate the over-production of nitric oxide and oxidative stress and protect neurons against Aβ_1–42_ induced neurotoxicity [18], suggesting potential relevance for AD treatment.

In rodent models, ZL006 reduced nitric oxide-mediated neurotoxicity and neuronal damage in conditions such as ischemia and traumatic brain injury (TBI) [19]. More recent studies in murine models demonstrated that ZL006 reduced aggressive behavior and nitric oxide levels in the brain of treated animals [20], along with alleviating thalamic pain through the disruption of nNOS/PSD-95 interaction. However, despite these promising findings, the use of ZL006 in mammalian models of AD remains limited and has not been systematically investigated. In this context, the zebrafish model offers several complementary advantages, and it also aligns with the 3Rs principle (Replacement, Reduction, Refinement). Its high fecundity (even more than 100 embryos per spawning) reduces the need for adult animals, enabling the employment of large numbers of embryos. According to the EU Directive 2010/63/EU18, up to five days post-fertilization, zebrafish is ethically considered as an in vitro model. Other advantages in the use of zebrafish include high genetic and molecular conservation with humans, rapid embryonic development and embryo transparency during early developmental stages, allowing in vivo imaging of neurodegenerative processes and large-scale drug discovery studies [21], as well as neuroanatomical architecture [22,23]. Importantly, the presence of genes orthologous to the ones mainly involved in the onset of AD (namely *psen1* and *psen2*) make zebrafish a relevant model for studies involving neuroinflammation and neurodegenerative processes [24,25,26,27].

The aim of our study was to investigate, for the first time, the effects of ZL006 using an established zebrafish (*Danio rerio*) in vivo model of FAD.

We also decided to employ zebrafish, because previous research showed that it can be used as a valid experimental model to study AD [28]. To achieve our goal, embryos were microinjected with *psen1*-morpholino, an antisense oligonucleotide that blocks the exon 8 splice acceptor site, thereby leading to a partial retention of intron 7. The aberrant splicing and consequent inefficient protein synthesis induced by *psen1* knockdown via morpholino generate truncated or dysfunctional proteins that can interfere with the function of the endogenous protein, thereby producing a dominant negative effect. *Psen1*-morpholino and mutant of zebrafish embryos at 4–5 days post fertilization (dpf) presented cognitive deficits, increased brain Aβ_1–42_ and ROS accumulation, along with decreased expression of synaptic marker PSD-95 [24,29,30,31,32,33]. We hypothesized that ZL006 treatment could exert a neuroprotective role in *psen1*-morphants by reducing neuroinflammation, oxidative stress and brain Aβ_1–42_ accumulation. Overall, our findings may lay the groundwork for further investigation into the neuroprotective and potential therapeutic properties of this compound for FAD patients.

## 2. Results

### 2.1. Zebrafish FAD Model

**Figure 1:**
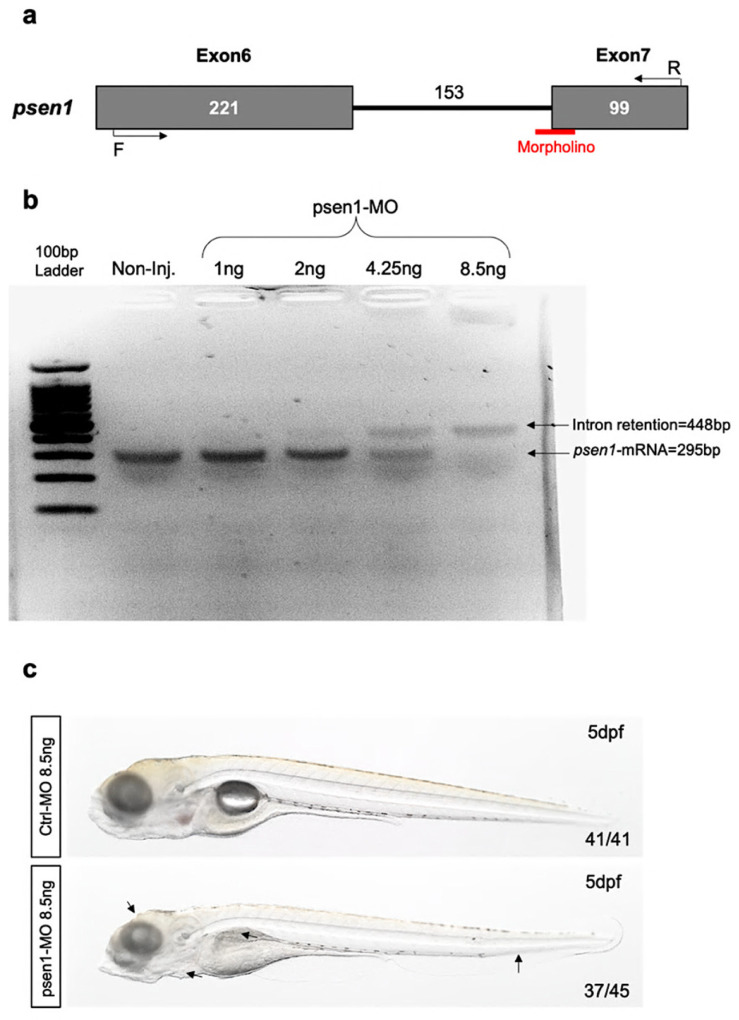
(**a**) Schematic of MO-retention targeting intron/exon7 junctions in *psen1*. Validation of the *psen1*-MO that induces intron retention. (**b**) Agarose gel of the RT-PCR was performed on mRNA/cDNA obtained at 48 hpf from pools of 15 embryos injected with *psen1*-MO at different concentrations (1; 2; 4.25; 8.5 ng) and non-injected. (**c**) Brightfield imaging of zebrafish embryos injected with ctrl-MO and *psen1*-MO 8.5 ng at 5 dpf. Black arrows indicate the regions in which morphological defects have been described in *psen1*-morphants; from left to right: head, heart, swim bladder and tail.

**Figure 2:**
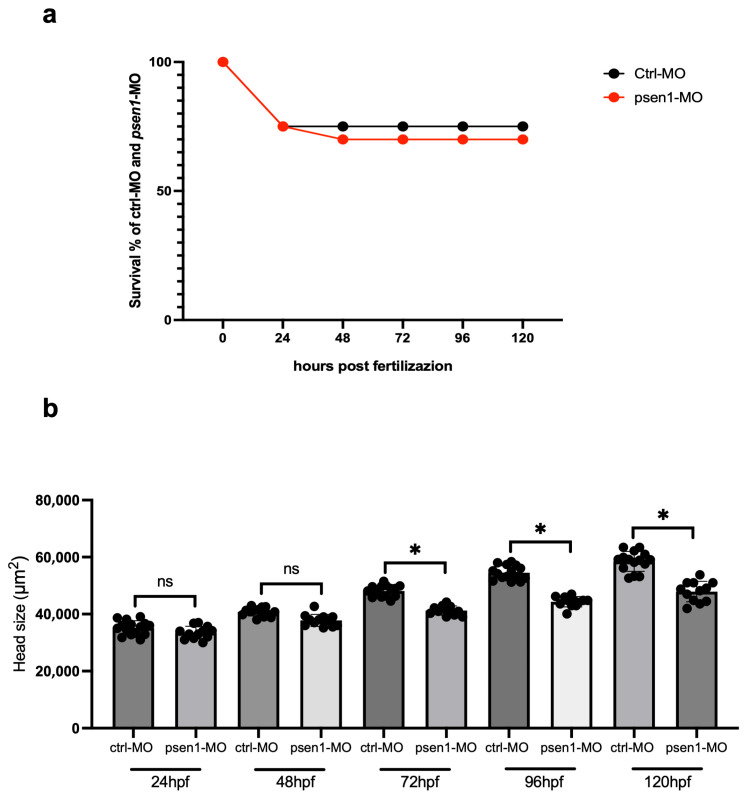
(**a**) Survival percentage of zebrafish embryo after injection of control and *psen1*-morpholinos. (**b**) Statistical analysis of head area, unpaired two-tailed *t* test. Center values denote the mean, and error values denote s.e.m. (ns = 0.45; ns = 0.31; * *p* < 0.01).

To investigate the effect of ZL006 in the zebrafish FAD model, we used a *psen1*-morpholino previously validated by Nornes and colleagues [24]. We injected *psen1*-morpholino at different concentrations (1; 2; 4.25; 8.5 ng). Our results are consistent with previous published data, showing that *psen1*-morpholino (intron/exon 7 junction) induced (an intron retention) the partial retention of intron 7 (Figure 1a,b). We also confirmed the morphological defects in the head, as well as swim bladder absence and defects at the level of the heart (edema) and tail (Figure 1c), consistent with what was also reported by Nornes and colleagues. Next, we monitored the survival up to five days post-fertilization (dpf), and we found no significant differences among groups injected with control morpholino (ctrl-MO) and morpholino targeting *psen1* (*psen1*-MO) (Figure 2).

Next, we monitored the survival up to 5 days post-fertilization (dpf), and we found no significant differences among groups injected with control morpholino (ctrl-MO) and morpholino targeting *psen1* (*psen1*-MO) (Figure 2a). We also analyzed the head size, comparing the control and *psen1*-morphants. We found a significant decrease in the head size in *psen1*-morphants from 72 to 120 hpf (Figure 2b). We validated *psen1*-morpholino injecting *psen1*-full length-mRNA. We observed a rescue of the morphology defects and head size in embryos co-injected with *psen1*-morpholino and *psen1*-*mRNA* (Supplementary Figure S1a,b).

### 2.2. Survival and Monitoring of Zebrafish Embryos Treated with ZL006

**Figure 3:**
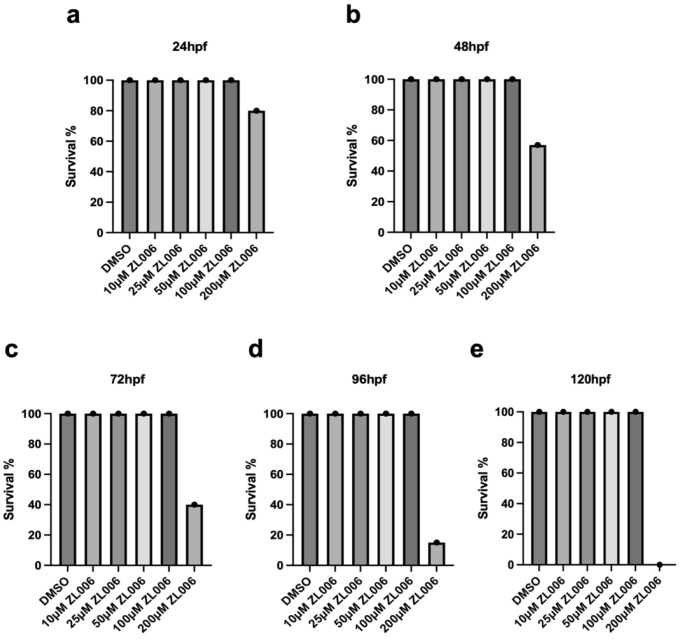
Survival rate of zebrafish embryos (30 embryos for each group) treated with DMSO and with different concentrations of ZL006 (10, 25, 50, 100, 200 μM) at 24 hpf (**a**), 48 hpf (**b**), 72 hpf (**c**), 96 hpf (**d**), 120 hpf (**e**).

To identify a non-lethal dose of ZL006 to treat zebrafish embryos, first we collected 180 embryos at 1 hpf, and we treated them with DMSO (control) and/or supplemented with ZL006 at different concentrations (10, 25, 50, 100, 200 μM) up to 120 hpf, respectively (Figure 3a–d). The embryo survival was monitored every 24 h. The survival rate is reported as the percentage of dead fish at 120 hpf compared to the control (DMSO group). As shown in Figure 2, we observed a significant decrease in the survival of embryos treated with a high concentration of ZL006 (200 μM), while ZL006 concentrations from 10 to 100 μM did not affect embryo survival (Figure 3a–e). For the next experiments, we chose to treat embryos with ZL006 100 μM, (as this specific concentration turned out to ameliorate the neurotoxic effects caused by the accumulation of Aβ_1–42_ in the *psen1*-morphant model, as detailed in the following analyses).

### 2.3. ZL006 Treatment Suppresses the Accumulation of Aβ_1–42_ in psen1 Morphants

**Figure 4:**
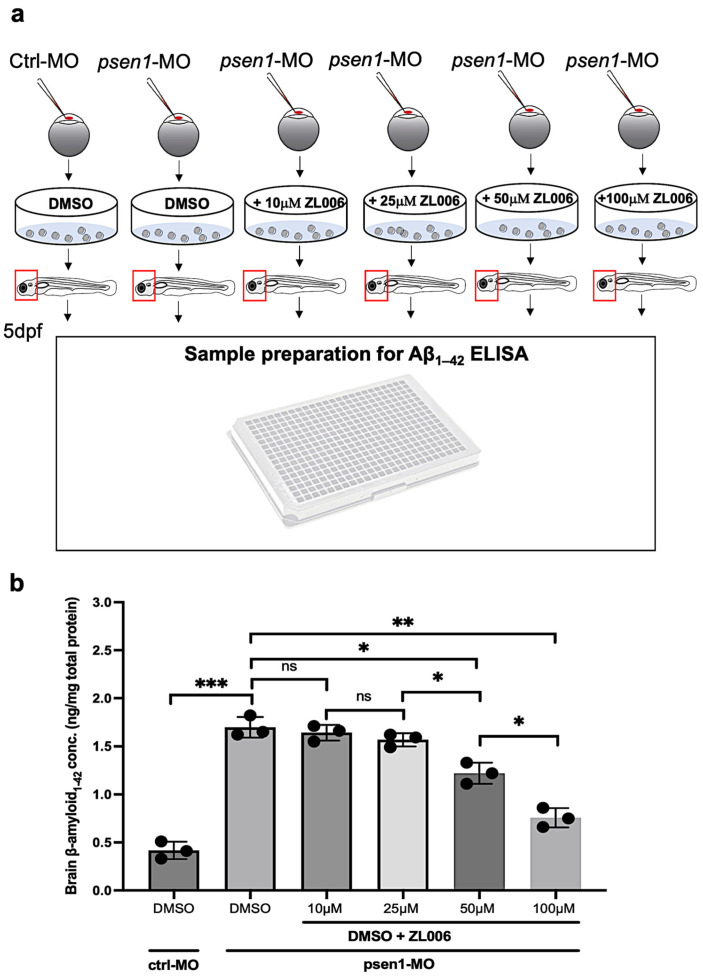
(**a**) Experimental outline to measure the Aβ_1–42_ level by ELISA kit in control and *psen1*-MO treated with DMSO and/or ZL006 at different doses (10, 25, 50, 100 μM) at 5 dpf.; red boxes highlight the head region used for the Aβ_142_ measurements. (**b**) Quantification of Aβ_1–42_ concentration in *psen1*-MO treated with DMSO and/or ZL006. Three biological replicates (180 embryos in total). Statistical analysis: one-way ANOVA with Tukey–Kramer post hoc tests, adjusted for multiple comparisons, *** *p*  <  0.0001; ** *p*  <  0.001; * *p*  <  0.01; ns = 0.42; ns = 0.63. Center values denote the mean, and error bars denote s.e.m.

Based on previously published results, the deficiency of *psen1* in zebrafish embryos induces an accumulation of β-amyloid (Aβ) in the brain [31]. As we mentioned before, a recent in vitro study showed that ZL006 exposure can protect neurons against the accumulation of Aβ_1–42_. To verify this hypothesis in our zebrafish model, we decided to evaluate the Aβ_1–42_ accumulation levels in control and *psen1*-morphants after treatment with DMSO and ZL006 at different doses (10, 25, 50, 100 μM). We injected wild-type AB* embryos with control- and *psen1*-morpholinos, and at 5 dpf, we processed the dissected head embryos and measured the Aβ_1–42_ levels using a specific ELISA assay, also described in a recent study [34] (Figure 4a). Through these analyses, we found that *psen1*-morpholino in embryos treated with DMSO induced a significant increase in Aβ_1–42_ concentration (Figure 4b). In *psen1*-morphants embryos treated with ZL006 (50 and 100 μM), we detected a significant decrease in Aβ_1–42_ levels. Consequently, our data show that ZL006 can suppress the accumulation of Aβ_1–42_ in a zebrafish FAD model. We can rescue the accumulation of Aβ_1–42_ by co-injecting *psen1*-morpholino and *psen1-mRNA* in embryos (Supplementary Figure S2a).

### 2.4. ZL006 Treatment Reduced the Oxidative Stress and Inflammation in psen1-Morphants

**Figure 5:**
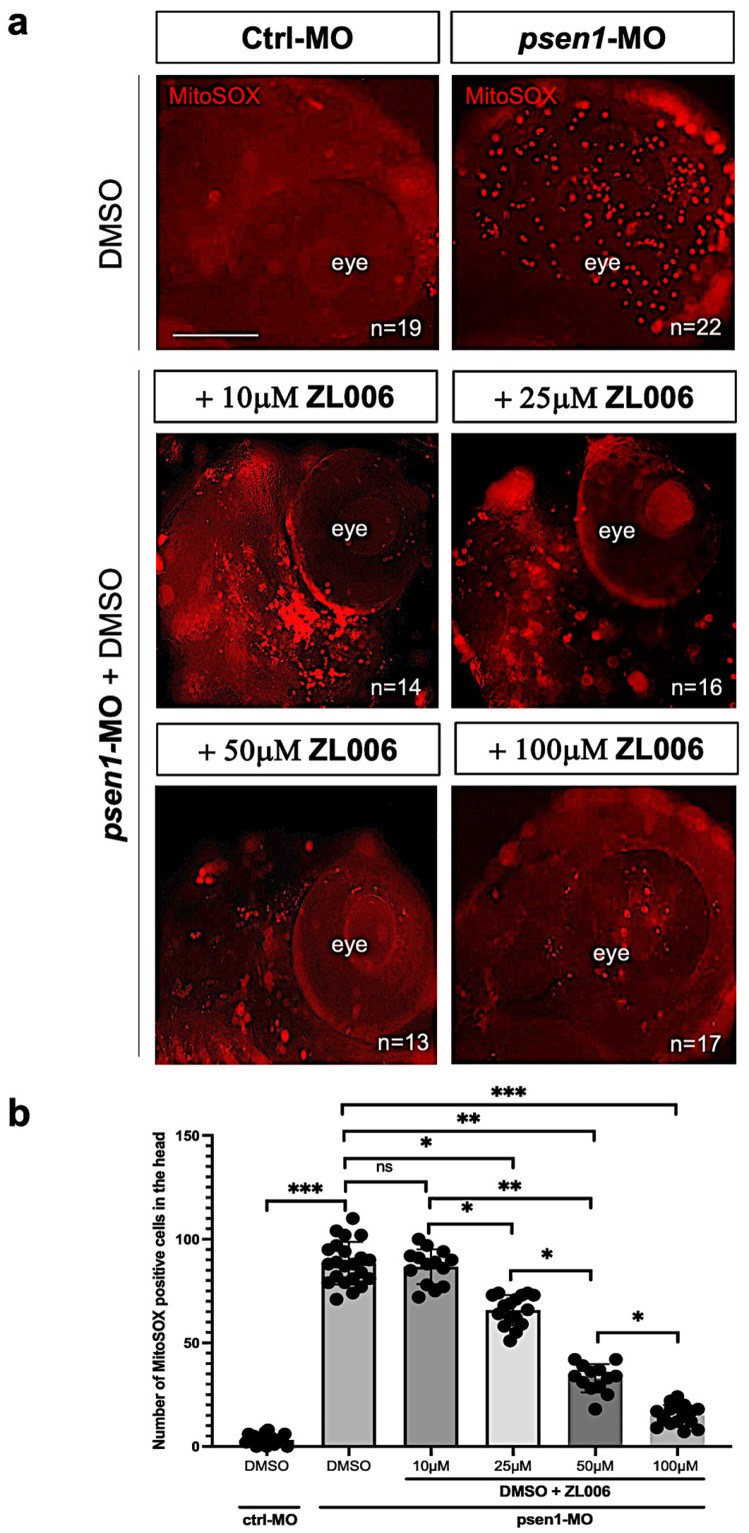
(**a**) Confocal imaging to detect MitoSOX fluorescent probe (red) in the head of control and *psen1*-morphants after treatment with DMSO and ZL006 (10, 25, 50, 100 μM). (**b**) Quantification of MitoSOX positive cells affected by oxidative stress in the head of *psen1*-morphants (*psen1*-MO) and control (ctrl-MO) at 5 dpf after treatment with DMSO and ZL006. The center values of all statistical analyses denote the mean, and error values denote s.e.m. Statistical analysis: one-way ANOVA with Tukey–Kramer post hoc tests, adjusted for multiple comparisons, *** *p*  <  0.0001; ** *p*  <  0.001; * *p*  <  0.01; ns = 0.73. Scale bar: 100 μm (**a**).

Oxidative stress plays a key role in the progression of FAD. In detail, previous studies showed that the accumulation of Aβ_1–42_ in neuronal mitochondria will damage the mitochondrial function by increasing ROS production and decreasing adenosine triphosphate (ATP) production [13]. ZL006 is a potent inhibitor of nNOS/PSD95-interaction, and it has been used to reduce over-produced nitric oxide and oxidative stress [17,18]. To verify this potential role in our zebrafish model, we decided to evaluate mitochondrial ROS accumulation in control and *psen1*-morphants after treatment with DMSO and ZL006 at different doses (10, 25, 50, 100 μM). We injected wild-type AB* embryos with control- and *psen1*-morpholinos, and we treated the embryos using DMSO and ZL006 up to 5 dpf. Next, we added MitoSOX probe in vivo to detect the oxidative stress levels. We found that *psen1*-morpholino induced a significant increase in MitoSOX-positive cells in the head of embryos treated with DMSO (Figure 5a,b). Finally, we found in *psen1*-morphants a significant decrease in MitoSOX-positive cells in the head of embryos treated with ZL006 at 25, 50 and 100 μM (Figure 5a,b). Our data confirm for the first time that ZL006 can reduce oxidative stress in a zebrafish FAD model.

**Figure 6:**
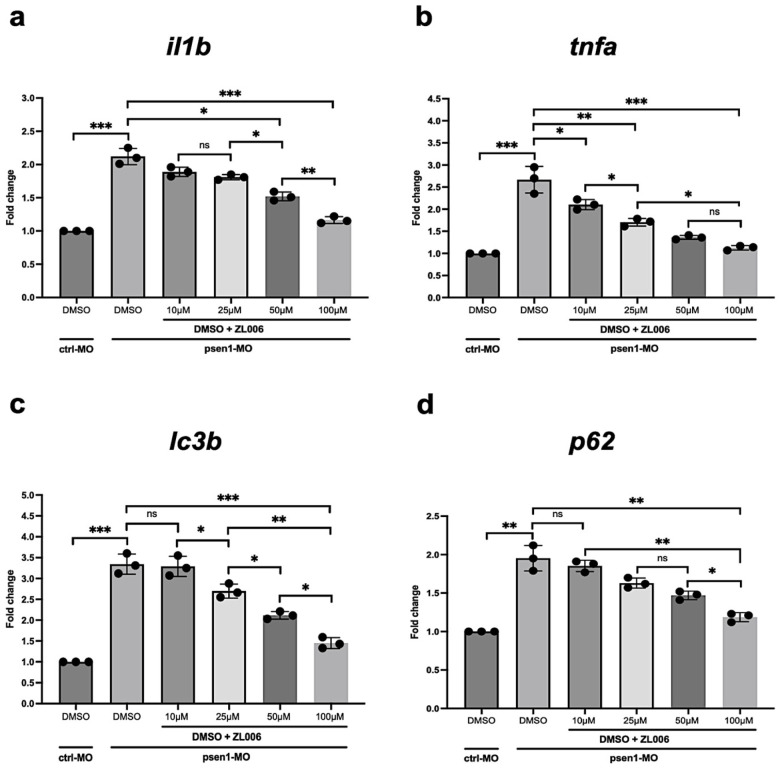
Quantitative real time PCR for different genes in the head of *psen1*-morphants (*psen1*-MO) and control (ctrl-MO) at 5 dpf after treatment with DMSO and ZL006 (10, 25, 50, 100 μM). (**a**) qPCR for *il1b* *** *p*  <  0.0001; ** *p*  <  0.001; * *p*  <  0.01; ns = 0.67. (**b**) qPCR for *tnfa* *** *p*  <  0.0001; ** *p*  <  0.001; * *p*  <  0.01; ns = 0.51. (**c**) qPCR for *lc3b* *** *p*  <  0.0001; ** *p*  <  0.001; * *p*  <  0.01; ns = 0.82. (**d**) qPCR for *p62* ** *p*  <  0.001; * *p*  <  0.01; ns = 0.72; ns = 028. Statistical analysis: one-way ANOVA, with Tukey–Kramer post hoc tests, adjusted for multiple comparisons. The center values of all statistical analyses denote the mean, and error values denote s.e.m.

In addition, we also analyzed the transcript level of pro-inflammatory cytokines *il1b* and *tnfa* and autophagy genes *lc3b* and *p62* by using qPCR. We found a significant reduction in *il1b, tnfa*, *lc3b* and *p62* in *psen1*-MO embryos treated with ZL006. In detail, we found that ZL006 doses at 50 and 100 μM were able to reduce inflammation and rescue autophagic flux in embryos injected with *psen1*-morpholino (Figure 6a,b). These phenotypes can be rescued by co-injecting *psen1*-morpholino and *psen1-mRNA* in embryos (Supplementary Figures S2b,c and S3a,b).

### 2.5. ZL006 Treatment Rescued the Loss of PSD95 Synaptic Marker and Reduced nNOS Level in psen1-Morphants

**Figure 7:**
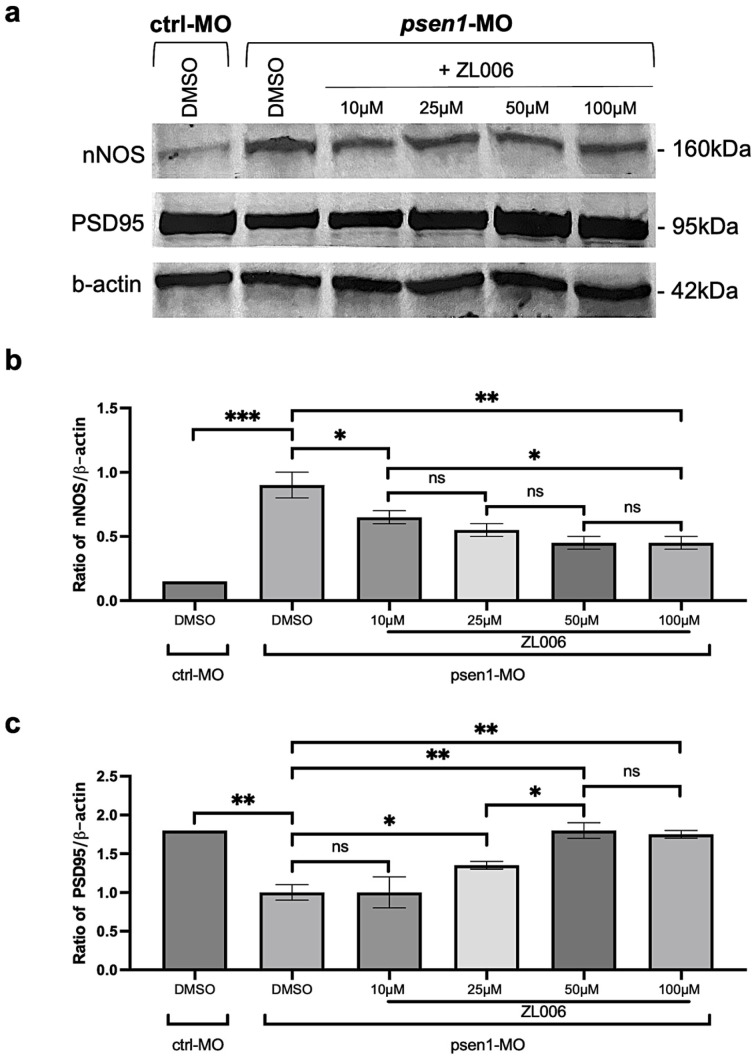
(**a**) Western blot analysis of extracts prepared from the head of control and *psen1*-morphants after treatment with DMSO and ZL006 (10, 25, 50, 100 μM) at 5 dpf shows a significant increase in nNOS protein (160 kDa) in *psen1*-morphants treated only with DMSO. ZL006 treatment was able to reduce the level of nNOS protein presented in zebrafish embryos injected with *psen1*-morpholino. *Psen1*-morphants (DMSO treated) also presented a significant reduction in PSD-95 protein. ZL006 at 25, 50 and 100 μM doses was able to rescue the loss of PSD-95. (**b**) Statistical analysis of the ratio nNOS/b-actin was completed using one-way ANOVA with Tukey–Kramer post hoc tests, adjusted for multiple comparisons. *** *p*  <  0.0001; ** *p*  <  0.001; * *p*  <  0.01; ns = 0.44; ns = 0.29; ns = 62. (**c**) Statistical analysis of the ratio PSD95/b-actin was completed using one-way ANOVA with Tukey–Kramer post hoc tests, adjusted for multiple comparisons. ** *p*  <  0.001; * *p*  <  0.01; ns = 0.62; ns = 0.71. The center values of all statistical analyses denote the mean, and error values denote s.e.m.

Based on previous studies, *psen1*-deficient zebrafish brain embryos present a loss of the synaptic marker PSD95, also known as *dlg4b* (ZDB-GENE-040628-3) [31], and a significant increase in nNOS. As we mentioned before, ZL006 is a potent inhibitor of nNOS/PSD-95 interaction. Based on this consideration, we mainly investigated the effects of this drug on nNOS and PSD-95 protein expression in our experimental zebrafish model. We injected wild-type AB* embryos with control- and *psen1*-morpholinos, and we treated the embryos with DMSO and different doses of ZL006 (10, 25, 50, 100 μM) up to 5 dpf. Next, we performed Western blot to measure the PSD-95 and nNOS protein levels (Figure 7a–c and Supplementary Figures S4–S6).

*Psen1*-morphant shows a significant increase in nNOS protein (160 kDa) in *psen1*-morphants treated only with DMSO. ZL006 treatment was able to reduce the level of nNOS protein in zebrafish embryos injected with *psen1*-morpholino (Figure 7a,b). *Psen1*-morphants (DMSO treated) also presented a significant reduction in the PSD-95 protein. ZL006 at 25, 50 and 100 μM doses was able to rescue the loss of PSD-95 (Figure 7a–c). Finally, we confirmed the previous results, performing in situ hybridization for *dlg4b* on brain transversal paraffin sections at 5 dpf.

**Figure 8:**
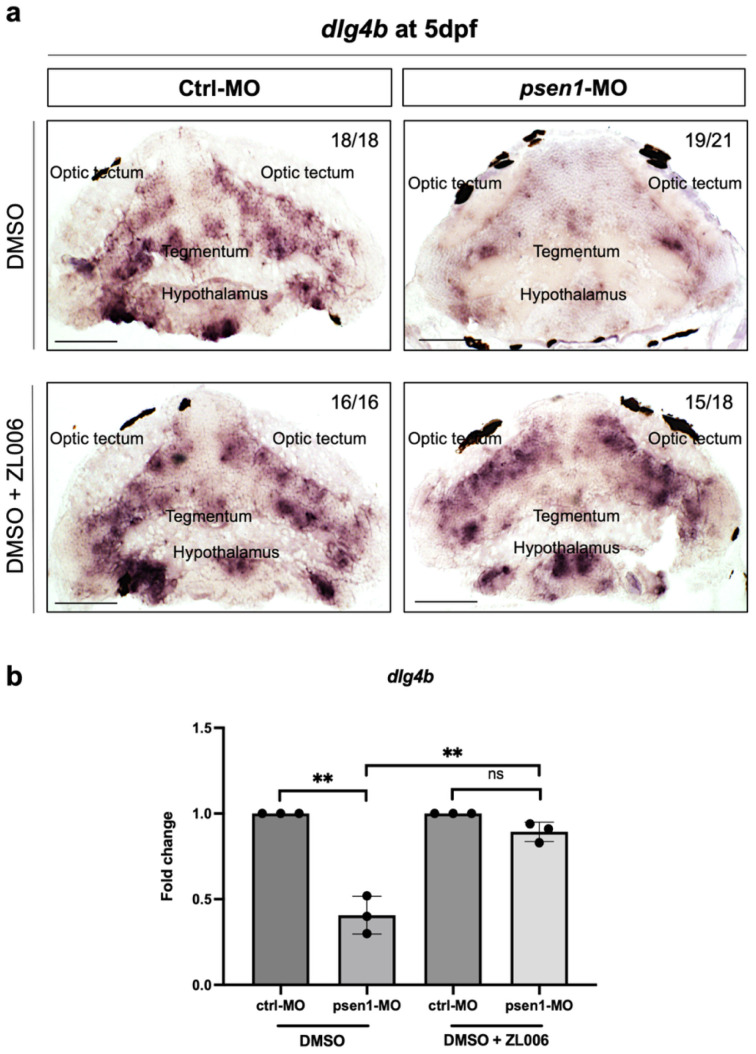
(**a**) In situ hybridization for *dlg4b* on paraffin transversal sections of zebrafish brain embryos injected with control and *psen1*-morpholino at 5 dpf treated with DMSO and/or ZL006. All the brain sections from ctrl-MO treated with DMSO and DMSO + ZL006 (18/18 and 16/16, respectively) showed normal *dlg4b* distribution; 19/21 brain sections from *psen1*-MO exposed to DMSO displayed reduced *dlg4b* expression; in *psen1*-morphants treated with DMSO + ZL006, 15/18 sections exhibited a rescue of *dlg4b* expression throughout the brain regions. Scale bar: 100 μm. (**b**) Quantitative real time PCR for *dlg4b* in the head of *psen1*-morphants (*psen1*-MO) and control (ctrl-MO) at 5 dpf after treatment with DMSO and ZL006. Statistical analysis: one-way ANOVA, with Tukey–Kramer post hoc tests, adjusted for multiple comparisons. ** *p*  <  0.001; ns = 0.15. The center values of all statistical analyses denote the mean, and error values denote s.e.m.

We observed a reduction in the *dlg4b* transcript in different brain regions (optic tectum, tegmentum and hypothalamus) of *psen1*-morphants (treated with DMSO). Interestingly, we found a rescue of *dlg4b* distribution in the brain embryo of *psen1*-morphants treated with DMSO and ZL006 (Figure 8a). To confirm our morphological observation, we used qPCR to quantify the transcription level of *dlg4b*. We found a significant decrease in *dlg4b* in *psen1*-morphants (treated with DMSO) compared to embryos injected with control-morpholino. Finally, we found a rescue of the *dlg4b* transcription level in *psen1*-morphants (treated with DMSO and ZL006) (Figure 8b).

## 3. Discussion

In the present study, we used a morpholino-based strategy previously validated in a published study [24]. In our results, we confirm the intron retention to exon 7 caused by *psen1*-morpholino. We also described phenotypic effects in terms of morphological defects also reported by Nornes and colleagues. The morphological effects of *psen1*-MO could be attributed to several pathways modulated by presenilin. Previous studies showed that *psen1*-deficiency can regulate the accumulation or degradation in β-catenin, a central factor of the Wnt signal pathway. In addition, *psen1* is also involved in the Notch pathway [35,36,37]. In detail, previous in vitro and in vivo studies showed that Psen1, Psen2, nicastrin (NCT), anterior pharynx defective 1 (APH1a or APH1b), and presenilin enhancer 2 form active γ-secretase complexes in cellular membranes. These complexes are responsible for the cleavage of single pass trans-membrane proteins such as Notch [38,39]. This hypothesis has been demonstrated by Nery and colleagues using the zebrafish model [31]. The authors showed that the injection of *psen1*-morpholino in zebrafish embryos caused a significant increase in *neurogenin 1*, a transcription factor negatively regulated by Notch, at the brain level of larvae at 5 dpf.

All these studies confirm that zebrafish is a valid experimental model to study FAD and can be used for drug discovery. In this study, we particularly take advantage of the main advances that zebrafish offers to investigate the potential neuroprotective role of ZL006 in *psen1*-morphant. Interestingly, a recent in vitro study showed that ZL006, a potent inhibitor of nNOS/PSD-95 interaction, could be used to reduce the excess of nitric oxide and oxidative stress to protect neurons against Aβ_1–42_-induced neurotoxicity [17,18,20], whose accumulation in the brain is a major AD-like hallmark [40,41]. The deficiency of *psen1* in mammal and zebrafish models induces an accumulation of β-amyloid (Aβ) in the brain [6,8,31,42,43]. For these reasons, we decided to verify whether ZL006 was able to reduce the accumulation of Aβ_1–42_. Based on survival and monitoring after administration of several *ZL006* concentrations on zebrafish embryos, we found that (10, 25, 50 and 100 μM) are non-lethal doses that could give rise to a specific phenotype able to reduce neurotoxicity due to Aβ_1–42_ and ROS accumulation. Next, we measured the level of Aβ_1–42_ by using an ELISA assay, dissecting the head of control and *psen1*-morphants after treatment with DMSO and/or ZL006. As expected, we found a significant increase in Aβ_1–42_ in *psen1*-morphants compared to control embryos treated with DMSO alone, reinforcing our model as a valid system for AD studies. We showed for the first time that ZL006 treatment at different doses can reduce the accumulation of Aβ_1–42_ in the brain of *psen1*-morphants. Our results suggest that this drug can protect AD brain from toxicity. As reported by several studies, a prominent and early feature of AD is represented by oxidative stress and inflammation [10,13,44,45]. As a matter of fact, the accumulation of Aβ_1–42_ in neuronal mitochondria damages the mitochondrial function by increasing ROS production and inflammation and decreasing adenosine triphosphate (ATP) production [46,47]. As mentioned before, ZL006 has been used to reduce over-produced nitric oxide and oxidative stress in Aβ_1–42_-treated neuronal cells. To assess whether this drug can affect nNOS/PSD-95 levels in our model, we performed Western blot analysis to measure nNOS and PSD-95 expression. The *psen1*-morphant shows a significant increase in the nNOS protein (160 kDa) in *psen1*-morphants treated only with DMSO. ZL006 treatment was able to reduce the level of nNOS protein presented in zebrafish embryos injected with *psen1*-morpholino (Figure 7a,b). *Psen1*-morphants (DMSO treated) also presented a significant reduction in the PSD-95 protein. ZL006 at 25, 50 and 100 μM doses was able to rescue the loss of PSD-95 (Figure 7a–c). Overall, our results indicate a reduction in nNOS levels and a rescue of PSD-95 expression following ZL006 treatment; however further investigations will be needed to clarify the disruption of nNOS/PSD-95 interaction or nNOS enzymatic activity in the zebrafish FAD model.

We used a fluorescent probe to detect the mitochondrial ROS level in the head of embryos injected with control and *psen1*-morpholino and treated with DMSO and/or ZL006. We found a significant increase in mitochondrial ROS levels in the head *psen1*-morphants treated with DMSO compared to control-MO. Differently, the administration of ZL006 (50 and 100 μM) reduced the mitochondrial ROS levels in embryos injected with *psen1*-morpholino. We also found that ZL006 (25, 50 and 100 μM) was able to suppress brain inflammation and restore autophagic flux in *psen1*-deficient embryos. These data reinforce the potential antioxidant function of ZL006. Finally, we confirmed previous results performing in situ hybridization for *psd95* (known as *dlg4b*) on brain transversal paraffin sections at 5 dpf. Previous studies showed that the AD brain presents a significant decrease in the number of synapses [48,49,50,51]. Through this analysis, we found a significant decrease in the *dlg4b* transcription levels in several brain regions of embryos injected with *psen1*-morpholino and treated with DMSO compared to control-MO. Of note, we observed the rescue of *dlg4b* expression in *psen1*-morphants treated with ZL006.

### 3.1. Limitations

Despite the valuable insights provided by this study, several limitations should be acknowledged. The morpholino-based approach used to generate the FAD zebrafish model is a well-established and widely adopted strategy for rapid gene-silencing studies during early development [24,31]. In our study, this approach enabled the efficient preliminary evaluation of the neuroprotective and anti-inflammatory effects of ZL006, yielding results consistent with observations reported in both in vitro and in vivo models [18,19]. Nevertheless, morpholino-mediated silencing is transient and may not fully reproduce the chronic pathological features associated with stable genetic models. Therefore, future studies will focus on the generation of a stable *psen1*-/- zebrafish mutant line to further validate the effects of ZL006 in a more robust and physiologically relevant FAD model. In addition, the stable mutant line will allow the implementation of behavioral and cognitive analyses, which were beyond the scope of the present study but are essential to comprehensively evaluate drug efficacy on functions primarily impaired in AD patients.

Another important limitation concerns the mechanistic interpretation of our findings. Although ZL006 treatment in *psen1*-MO embryos modulated the expression levels of nNOS and PSD-95, the present data do not directly demonstrate disruption of the nNOS/PSD-95 interaction or direct inhibition of nNOS activity in vivo. Therefore, our findings support an association between ZL006 treatment and the regulation of nNOS/PSD-95-related pathways rather than definitive mechanistic proof of pathway inhibition. To address this limitation, future studies will include direct mechanistic assays such as nitric oxide quantification and nNOS activity measurements. In particular, the colorimetric Griess assay will be employed to indirectly evaluate nitric oxide production through the detection of extracellular nitrite, a stable degradation product of nitric oxide.

### 3.2. Conclusions

In conclusion, our study shows, for the first time, that ZL006 drug treatment can reduce brain Aβ1–42 accumulation, oxidative stress, inflammation and rescue the autophagic flux and loss of PSD95 in an FAD-associated *psen1*-deficient zebrafish in vivo model.

## 4. Material and Methods

### 4.1. Zebrafish Husbandry

Fish were raised according to FELASA. Embryos were obtained as previously described [52]. The experiments were made to comply with the 3R guidelines. AB* zebrafish strains were kept in a 14/10 h light/dark cycle at 28 °C. No authorization was required, since all experiments were performed up to 5 days post fertilization.

### 4.2. Morpholino and Full-mRNA Injections

The *psen1*-morpholino oligonucleotide (*psen1*-MO) and control-MO (ctrl-MO) were purchased from GeneTools (Philomath, OR, USA). The control-MO and *psen1*-MO sequences are listed in Table 1. *Psen1*-MO was validated in a previous study [24] (*psen1* in ensembl ENSDART00000149864.3). We confirmed the intron retention by reverse transcription-polymerase chain reaction (RT-PCR) from total RNA extracted from ∼10 embryos at 48 hpf using primers listed in Table 1.

*Psen1*-mRNA was reverse transcribed using a mMessage Machine kit SP6 (Ambion Austin, TX, USA) from a linearized pCS2+ vector containing PCR-amplified product. After transcription, RNA was purified by phenol–chloroform extraction. The oligonucleotide primers used are listed in Table 2.

### 4.3. DMSO and ZL006 Embryo Treatment

Zebrafish embryos were treated from 1 h post-fertilization (hpf) up to 120 hpf with DMSO (0.2%) and ZL006 at different concentrations (10; 25; 50; 100; 200 μM) to define the survival rate and morphology defects. Only fertilized eggs with a normal developmental phase were used for the experiments. Every experiment was performed in triplicate.

### 4.4. Enzyme-Linked ImmunoSorbent Assay (ELISA) for Aβ_1–42_

The Aβ_1–42_ content was determined, as described in a recent study [34]; we used a specific zebrafish Aβ_1–42_ ELISA kit (202308, Shanghai Enzyme Linked Biotechnology Co., Ltd., Shanghai, China), according to the manufacturer’s instructions. Briefly, zebrafish at 5 dpf were euthanized by freezing them using liquid nitrogen. Cold physiological saline was added to the embryo in a 1.5 mL tube at a ratio of 1:9 (mass:volume), homogenized, and centrifuged at 5000× *g* for 10 min. The supernatant was collected for the assay. The absorbance was measured at 450 nm using a microplate reader. The Aβ_1–42_ concentrations were measured as ng/mg total protein.

### 4.5. Mitochondrial Reactive Oxygen Species (ROS) Detection

The detection of oxidative stress was performed on living zebrafish embryos at 120 hpf by using MitoSOX (Invitrogen, Waltham, MA, USA). Embryos were exposed to 5 μM of the MitoSOX solution for 30 min at 28 °C, followed by analysis using fluorescence microscopy, as previously described [53,54].

### 4.6. RNA Extraction and Reverse Transcription

Ten embryo heads for each condition at 5 days post-fertilization (dpf) were used to extract the total RNA. After head dissection, we pooled and dissociated by using an RNAeasy minikit (Qiagen, Frankfurt, Germany). Next, we followed the manufacturer’s protocol to obtain purified RNA. This procedure was repeated in three independent experiments (120 embryos in total). For reverse transcription into cDNA, 0.5 μg of total RNA was incubated with a buffer mix and enzyme using the Superscript III First-Strand Synthesis System kit (Invitrogen, Boston, MA, USA). In detail, 10 μL of the total volume was incubated for 10 min at 25 °C, 30 min at 50 °C, and 5 min at 85 °C. Next, the samples were treated with RNase-H (ThermoFisher Scientific, Waltham, MA, USA) for 30 min at 37 °C.

### 4.7. Quantitative Real-Time Polymerase Chain Reaction (qRT-PCR)

Quantitative RT-PCR experimental procedures were performed by using a thermocycler with a MyiQ detector (Bio-Rad, Hercules, Dallas, TX, USA). Briefly, we mixed cDNA with specific forward and reverse primers, SYBR-Green (Bio-Rad, Hercules, Dallas, TX, USA), and RNase-free water according to the manufacturer’s protocol. The previous mix was incubated for 15 min at 95 °C, for 15 s at 95 °C for 40 cycles, for 30 s at 60 °C for 40 cycles, and for 30 s at 72 °C for 40 cycles. The primer sequences for the PCR gene amplification are listed in Table 3. Data are represented as the fold change of *il1b*, *tnfa*, *lc3b*, *p62* and *dlg4b* mRNA levels in control and *psen1*-morphants treated with DMSO and/or ZL006, using *ef1a* to normalize the absolute quantification, calculated using 2^−∆∆Ct^. To confirm the correct amplification, we performed a melting curve analysis and verified the PCR’s efficiency. Each qRT-PCR experiment was performed using biological triplicates. In the qRT-PCR analyses, each n represents the average of biological triplicates from a single experiment. All experiments were repeated at least three times.

### 4.8. Chromogenic In Situ Hybridization (ISH)

Digoxigenin (DIG)-labeled antisense riboprobes were prepared as reported in previous studies [55,56]. In detail, *dlg4b* expression was detected (using) through in situ hybridization (ISH). To generate the *dlg4b* probe, the vector TOPO-TA was linearized holding the product amplified using the polymerase chain reaction (PCR). Next, to produce antisense and sense ribo-probes, the plasmids were linearized with the specific enzymes. After plasmid linearization, in vitro transcription was performed, using SP6 (Roche-Diagnostic, Chicago, IL, USA) and adding the label mix digoxigenin RNA. To generate *dlg4b* riboprobe, we used the primers listed in Table 4.

After the production of the specific riboprobes, embryos at 5 dpf were fixed in paraformaldehyde (PFA) 4%, and kept overnight at 4 °C. After 24 h, the embryos were processed for paraffin. The brain embryo sections (10 μM) were obtained using a rotary microtome and mounted on slides. We deparaffinized all sections through immersion in xylene two times (3 min) and rehydrated them in ethanol at 100%, 95%, 80%, 70%, and 50% (3 min each). To fix, the sections were immersed for 20 min in PFA 4%. Then, the embryo brain (embryo) sections were immersed in PBS, and we added the proteinase K diluted 2 mg/mL at room temperature for 7 min. Next, all slides were processed as follows: fixed in 4% paraformaldehyde for 20 min and washed in PBS and standard saline sodium citrate (SSC 2x) 2 times (10 min each). Next, the slides were incubated at 63 °C for 24 h, using a moist chamber with the probes (2 μg/mL) diluted in a specific medium (Denhart 5x; SSC 2x; 50% formamide; ethylenediamine-tetra acetic acid 4 mM; 5% dextran sulfate; yeast tRNA 50 μg/mL). After 24 h, the sections were washed with SCC 2x, 50% formamide/SCC 2x, SSC 0.2x and SSC 0.1x. The sections were then immersed in a (buffer) Tris-HCl/NaCl buffer (mixing 100 mM of Tris-HCl pH 7.5 and 150 mM NaCl) and washed in the same buffer containing 0.5% milk powder and adding 0.1% Triton.

The next day, all sections were incubated with anti-digoxigenin alkaline phosphatase Fab fragments, at a dilution of 1:5000 (Roche Diagnostic company, Chicago, IL, USA), overnight at room temperature (RT). After 24 h, all sections were washed in Tris-HCl/NaCl buffer and with 110 mM HCl-Tris (pH 8) containing 10 mM MgCl2 and 110 mM NaCl. Staining was performed using NBT/BCIP chromogen solution buffer (pH 9.5).

#### 4.8.1. Western Blot

Three independent experiments were conducted with 5 head embryos pooled for each condition (150 embryos in total). Cells were lysed in Pierce IP Lysis Buffer (Thermo Scientific, Waltham, MA, USA) with protease inhibitor cocktail (Merck, Darmstadt, Germany), and proteins were extracted. Proteins were separated by SDS gel electrophoresis and incubated overnight at 4 °C with nNOS (1:1000; GeneTex, Irvine, CA, USA; GTX133407); PSD-95 (1:1000; Abcam, Cambridge, UK) and b-actin (1:1000 Invitrogen, Carlsbad, CA, USA) followed by goat anti-rabbit IgG (H1L)-horseradish peroxidase-conjugated secondary antibody (1:5000; Bio-Rad, Hercules, CA, USA) and goat-anti-mouse IgG (H/L)-horseradish peroxidase-conjugated secondary antibody (Biorad; 1:5000). Staining was revealed by using Western Bright Sirius (1:1; Adventa, San Jose, CA, USA) with an exposure of 1 min and 15 s.

#### 4.8.2. Fiji Software Analysis

To quantify the MitoSOX-positive cells, we used the automated ImageJ free version 1 software and the specific platform tool for measurement analysis, also known as Fiji (version 2.9.0).

#### 4.8.3. Statistical Analysis

For statistical analysis we used GraphPad Prism version 10.4.1 software (GraphPad Inc., San Diego, CA, USA); the data are normally distributed, and we used one-way ANOVA with Tukey–Kramer post hoc tests, adjusted for multiple comparison.

## Figures and Tables

**Table 1:** Morpholinos and primer sequences.

control-MO	CCTCTTACCTCAGTTACAATTTATA
*psen1*-MO	ACGTCTTGAACACTTCCCTGGAGGG
*psen1*-Primer Forward	GATGGATTACTTCACGCTG
*psen1*-Primer Reverse	GAGTAGATGAGCGCTGG

**Table 2:** Primer sequences targeting the *psen1* full-length mRNA.

*Full-psen1*-Primer Forward	CAGTTCCGATGGCTGATTTAGT
*Full-psen1*-Primer Reverse	CCTCTCTATATGTAGAACTGATGGAC

**Table 3:** Primer sequences for qPCR.

*il1b*-Forward	5′-ATGGCGAACGTCATCCAAGA-3′
*il1b*-Reverse	5′-GAGACCCGCTGATCTCCTTG-3′
*tnfa*-Forward	5′-TCACGCTCCATAAGACCCAG-3′
*tnfa*-Reverse	5′-GATGTGCAAAGACACCTGGC-3′
*dlg4b*-Forward	5′-ATCCACGCATACACACCTCAG-3′
*dlg4b*-Reverse	5′-CAACATCTCCGTCCATACCGT 3′
*ef1a*-Forward	5′-CCTGGGAGTGAAACAGCTG-3′
*ef1a*-Reverse	5′-GCCTCCAGCATGTTGTCAC-3′
*p62*-Forward	5′-GCGTCAGTGAGGGAACAAAG-3′
*P62*-Reverse	5′-CAGAGACTCCACCAGCCTAG-3′
*lc3b*-Forward	5′-CCTCCAACTCAACTCCAACC-3′
*lc3b*-Reverse	5′-GCCGTCTTCGTCTCTTTCC-3′

**Table 4:** Primer sequences for *dlg4b* probe.

*dlg4b*-Forward	GCCTCTCAAACGAGAAGATAC
*dlg4b*-Reverse	CCTGCCGTCTGATTCTCAAA

## Data Availability

The original contributions presented in this study are included in the article/Supplementary Material. Further inquiries can be directed to the corresponding author(s).

## Supplementary Materials

The following supporting information can be downloaded at https://www.mdpi.com/article/10.3390/ijms27114992/s1.
