# Supplementary material for: ZL006 Treatment Reduces Inflammation, Oxidative Stress, and Brain Aβ1–42 Accumulation and Rescues the Loss of PSD95 Synaptic Marker in Familial Alzheimer’s Disease-Associated psen1-Deficient Zebrafish Model
Source: Int J Mol Sci. 2026 May 30;27(11):4992. doi: 10.3390/ijms27114992 (PMC13256367; doi:10.3390/ijms27114992)
Supplement: Supplementary file 1 [file ijms-27-04992-s001.zip › ijms-4265386-supplementary.pdf]

Supplementary figure 1

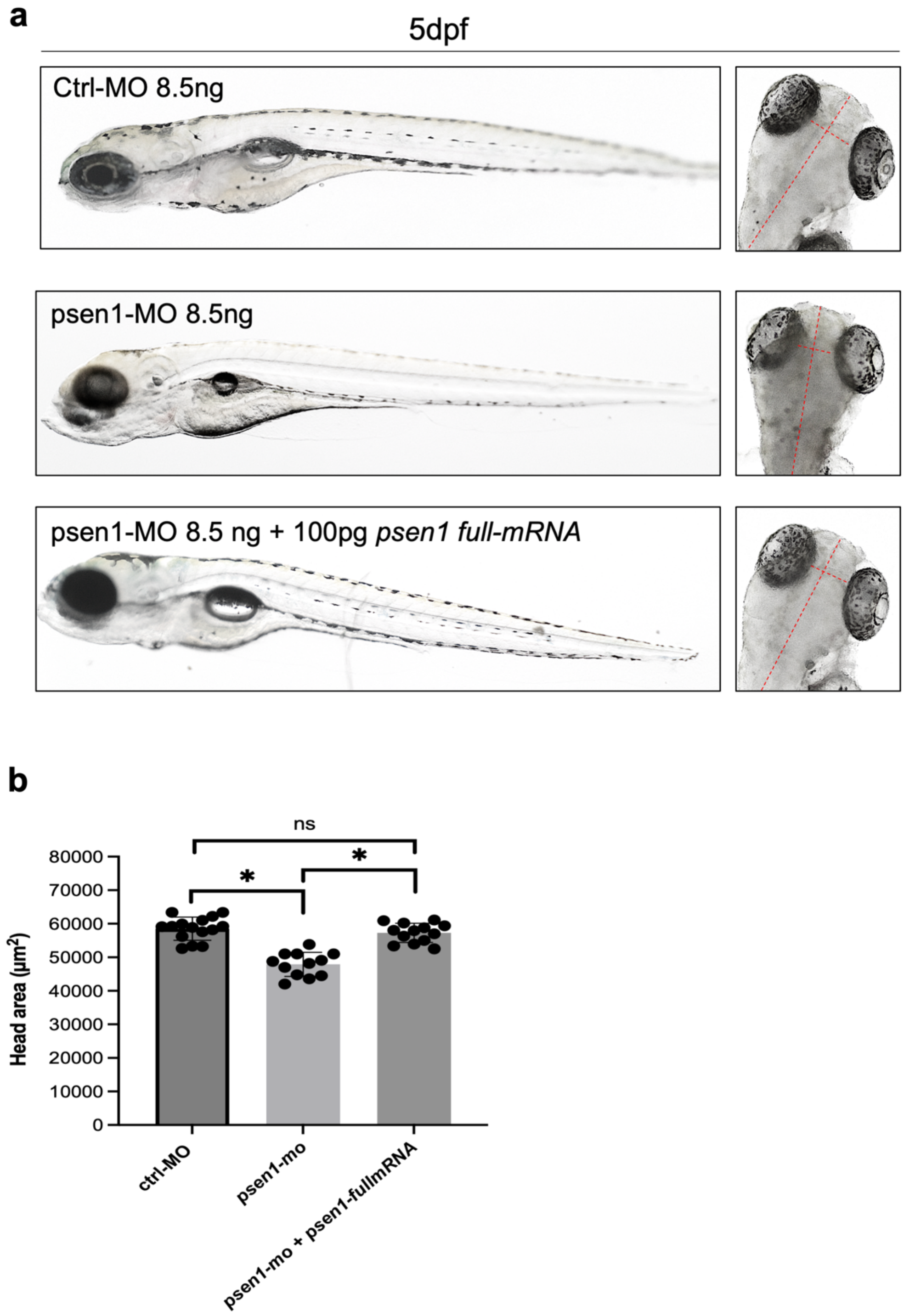

Supplementary figure 1. a) brightfield images of zebrafish embryos injected with control; psen1-morpholino and *psen1-mRNA* at 5dpf. b) Statistical analysis of head area, one-way ANOVA with Tukey–Kramer post hoc tests, adjusted for multiple comparisons. (ns= 0.68; $*p < 0.01$). Center values denote the mean, and error values denote s.e.m.

Supplementary figure 2

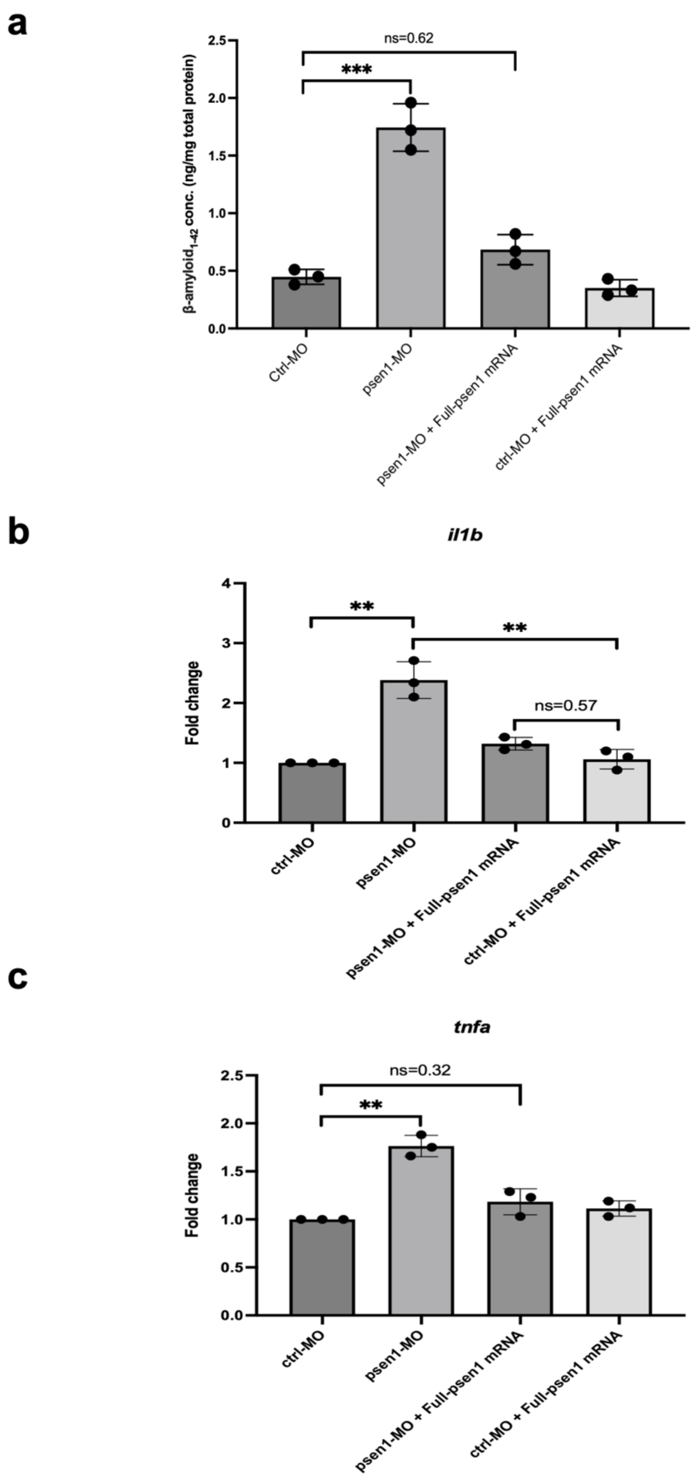

Supplementary figure 2. a) Quantification of Aβ_{1-42} concentration in ctrl, psen1-MO and embryo co-injected with *psen1-mRNA*. Quantitative real time PCR at 5 dpf for b) *il1b* and c) *tnfa* in the head of psen1-morphants (psen1-MO) and control (ctrl-MO) coinjected with *psen1-mRNA.* Statistical analysis: one-way ANOVA with Tukey–Kramer post hoc tests, adjusted for multiple comparisons.

Supplementary figure 3

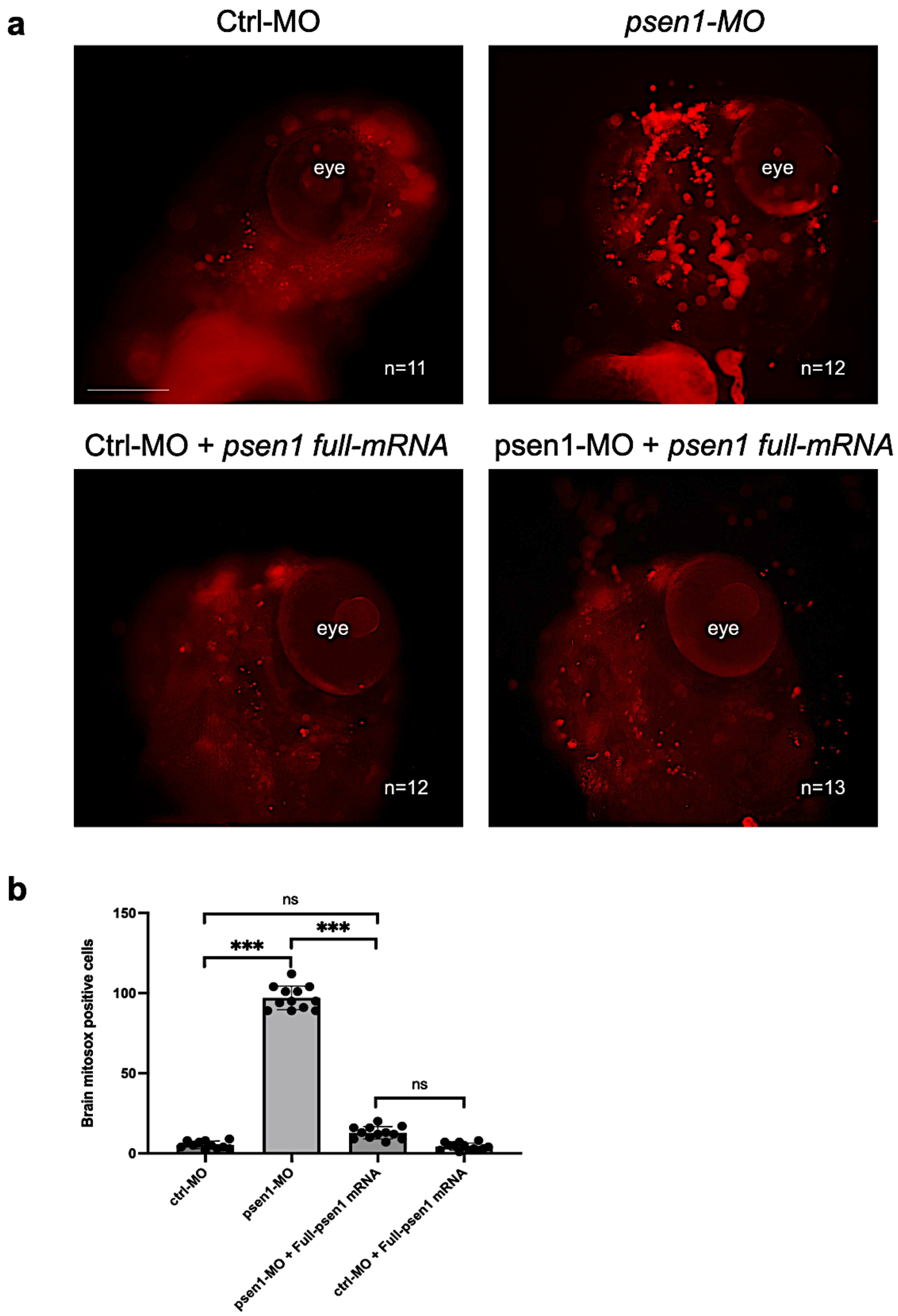

Supplementary figure 3. a) Confocal imaging to detect MitoSOX fluorescent probe (red) in the head of control and psen1-morphants and/or co-injected with psen1-mRNA. b) Quantification of MitoSOX positive cells affected by oxidative stress. The center values of all statistical analyses denote the mean, and error values denote s.e.m. Statistical analysis: one-way ANOVA with Tukey–Kramer post hoc tests, adjusted for multiple comparisons, ***$P < 0.0001$; ns=0.21 ; ns=0.33. Scale bar: 100µm (a).

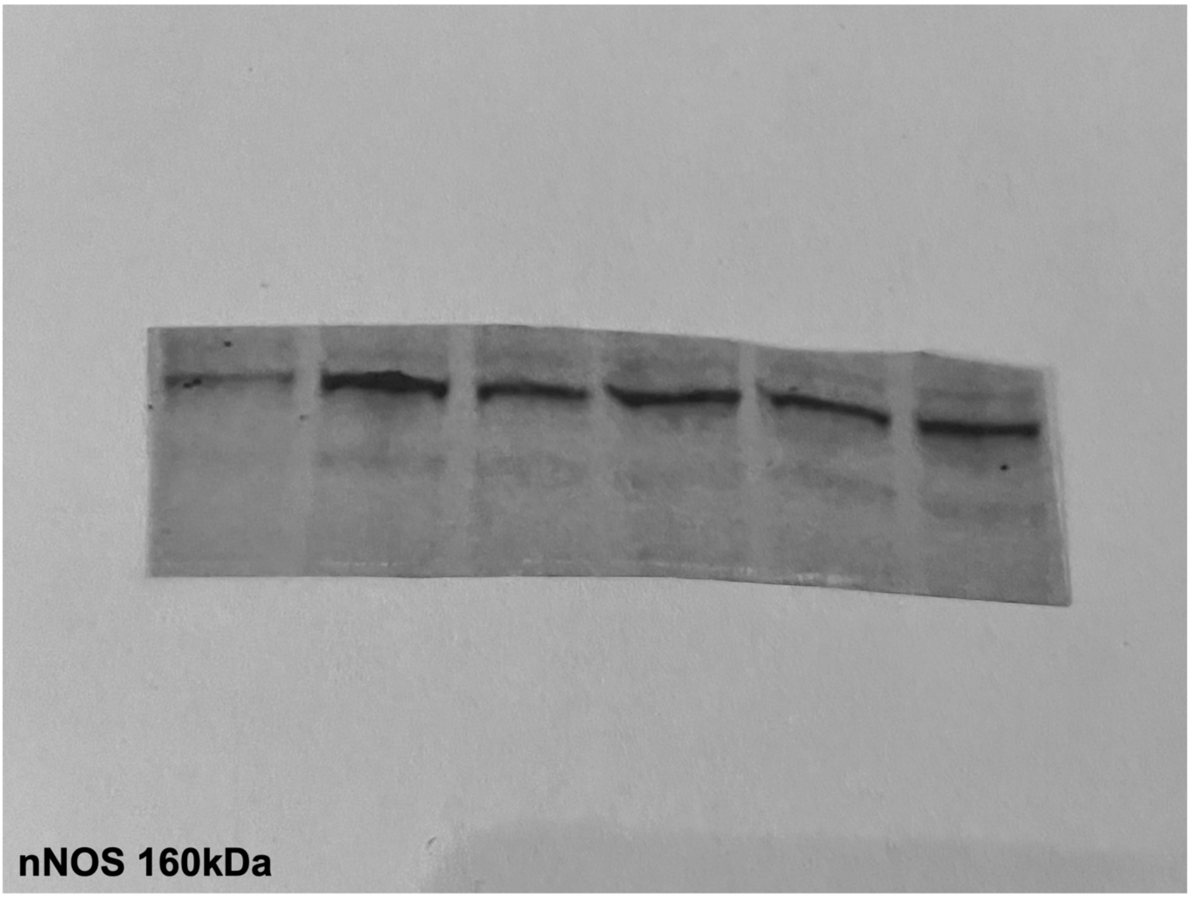

Supplementary figure 4. Western blot original uncropped for nNOS.

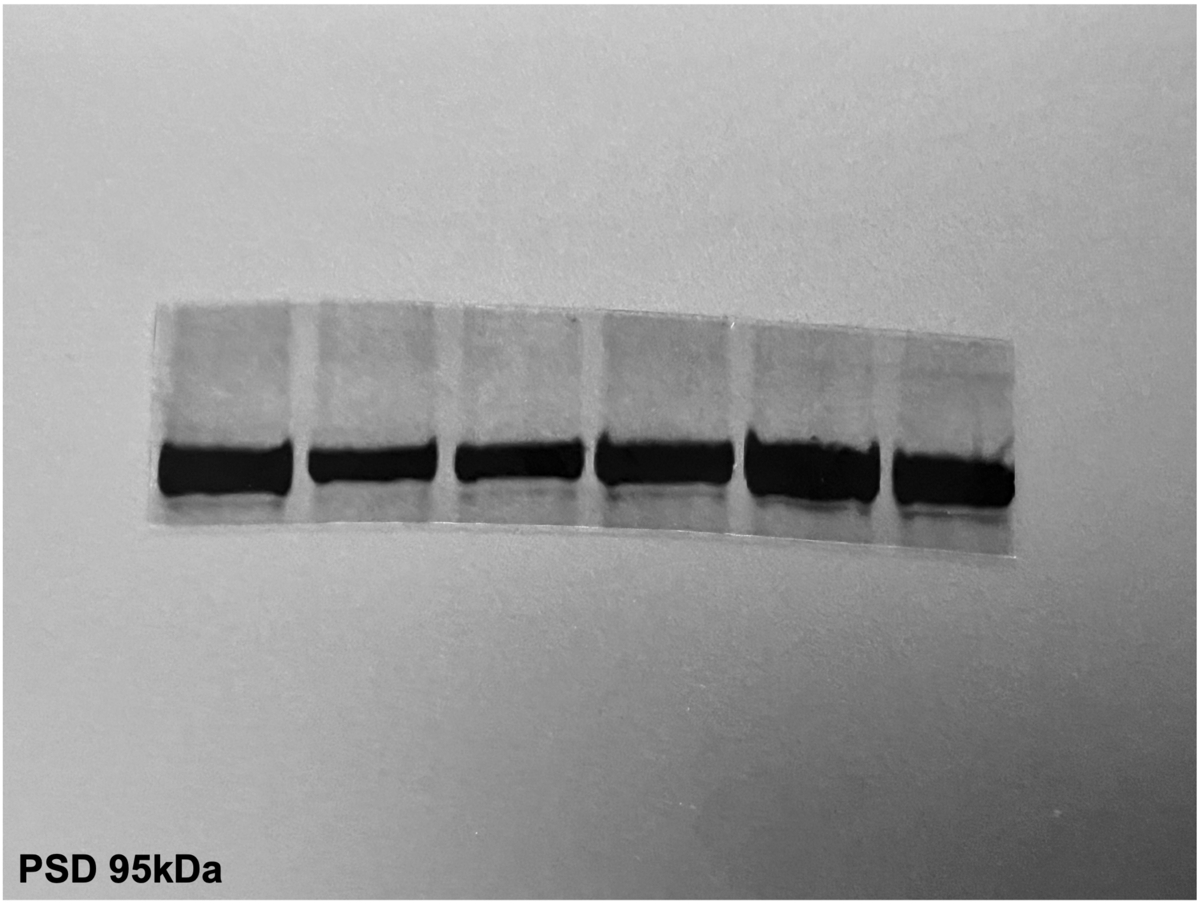

Supplementary figure 5. Western blot original uncropped for PSD95

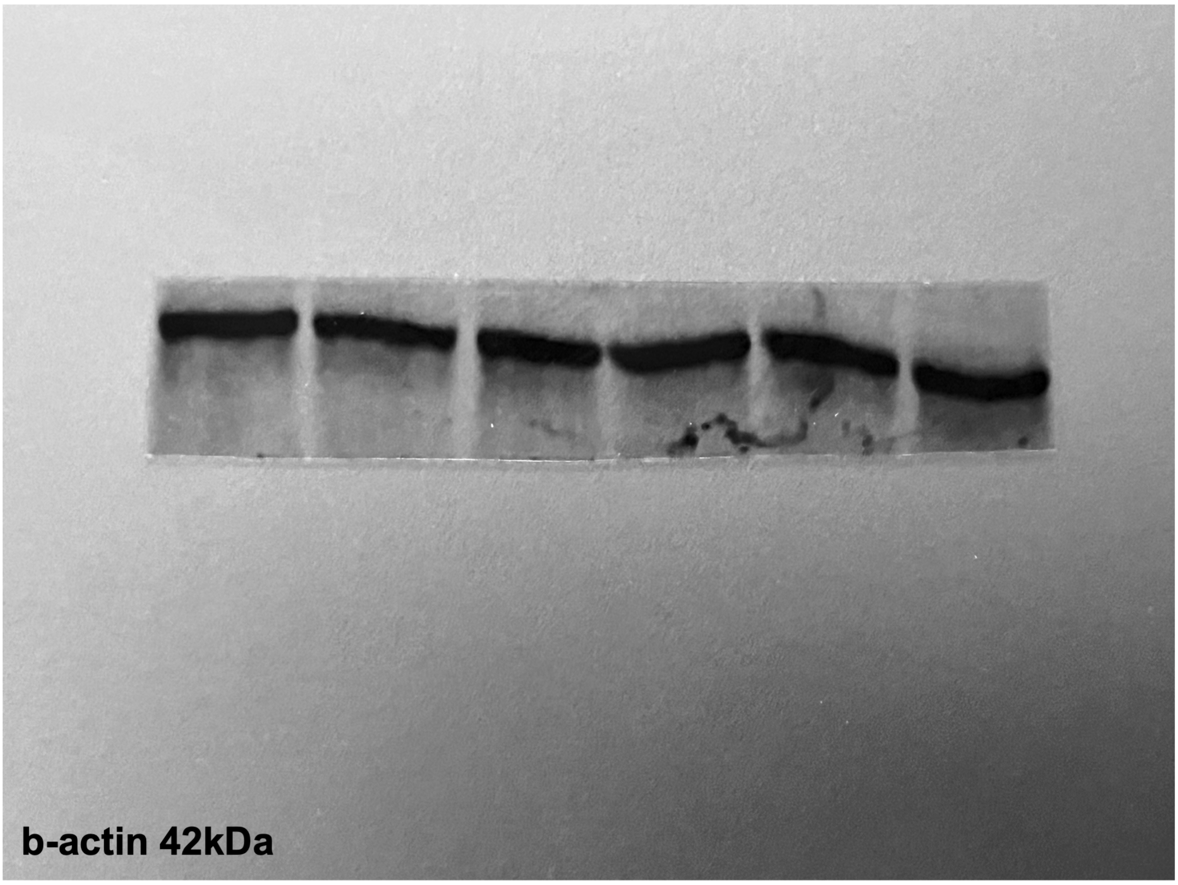

Supplementary figure 6. Western blot original uncropped for b-actin
